# Toothpick Aspiration Induces Massive Hemoptysis: a Case Report

**Published:** 2019-04

**Authors:** Mitra Rezaei, Najmeh Esfandiari, Seyed Reza Saghebi, Mihan Pourabdollah, Payam Tabarsi

**Affiliations:** 1 Virology Research Center, National Research Institute of Tuberculosis and Lung Diseases (NRITLD), Shahid Beheshti University of Medical Sciences, Tehran, Iran; 2 Department of Pathology, School of Medicine, Shahid Beheshti University of Medical Sciences, Tehran, Iran.; 3 Chronic Respiratory Diseases Research Center, NRITLD, Shahid Beheshti University of Medical Sciences, Tehran, Iran; 4 Lung Transplantation Research Center, NRITLD, Shahid Beheshti University of Medical Sciences, Tehran, Iran.; 5 Clinical Tuberculosis and Epidemiology Research Center, NRITLD, Shahid Beheshti University of Medical Sciences, Tehran, Iran.

**Keywords:** Massive hemoptysis, Toothpick, Foreign body aspiration, Ulceration

## Abstract

**Background:**

Massive hemoptysis refers to bleeding from the sputum exceeding 100 ml/day. This condition is known to have a poor prognosis. Although foreign body aspiration is not as common as other risk factors, it may result in massive hemoptysis. In the current study, we presented a case of massive hemoptysis due to the aspiration of a toothpick.

**Case Presentation:**

The patient was a 49-year-old woman who was primarily suspected of having tuberculosis. After observing blood in the sputum, interventions, including chest computed tomography (CT) scan and conservative management, were performed. The CT scan showed no malignancy, and paraclinical investigations were negative. However, hemoptysis was progressing into an acute phase; therefore, a surgical intervention was performed for the patient. After the surgery, the cause of the lesion was found to be a toothpick. The patient was under intensive care after surgery and was discharged from the hospital in a good general condition. The morphological evaluation of the lesion showed a bronchial wall with ulceration, besides granulation tissue formation, hematoma, and fibrinoid necrosis due to foreign body aspiration into the lung, resulting in inflammatory reactions.

**Conclusion:**

In this case report, foreign body aspiration resulted in massive hemoptysis. Our primary attempts to diagnose the cause of lesion were unsuccessful, and surgery was performed due to the life-threatening condition of the patient. Overall, unexplained hemoptysis may occur following a serious accident due to foreign body aspiration.

## INTRODUCTION

Hemoptysis is defined as the coughing up of blood. Although massive hemoptysis does not have an accepted definition, clinicians use this term when the volume of coughing blood exceeds 100 mL per day (even 1000 mL during a day of care) (1). According to the literature, the most common causes of hemoptysis are as follows: (I) infectious diseases, including active pulmonary tuberculosis, fungal infection, abscess, and necrotizing pneumonia due to *Klebsiella*, *Staphylococcus*, and *Legionella* species; (II) iatrogenic causes, such as catheterization, bronchoscopy, transbronchial biopsy, and transtracheal aspiration; (III) trauma due to suction ulcers and tracheoarterial or bronchovascular fistula; (IV) parasitic infections, such as hydatid cyst and paragonimiasis (the former is prevalent in Iran, unlike the latter); (V) cardiovascular problems; (VI) coagulation disorders due to coagulation factor disorders, platelet dysfunction, or thrombocytopenia; and (VII) and cystic fibrosis (2–5).

While the prognosis of hemoptysis is satisfactory in most cases, massive hemoptysis has a poor prognosis (3). Hemoptysis is an emergency condition, which requires emergency care, especially in severe life-threatening cases (4). However, foreign body aspiration is not as common as other risk factors, and most cases have been reported in children (6–9). In both children and adults, hemoptysis and bronchitis are usually common symptoms when the foreign body remains in the body for a long time after aspiration (10).

In the current report, we present a case of massive hemoptysis, which was initially misdiagnosed as tuberculosis (TB). We found that the cause of hemoptysis was the aspiration of a toothpick into the lung.

## CASE SUMMARIES

The patient was a 49-year-old housewife, who was a non-smoker, without a history of respiratory diseases. She was initially surveyed in a hospital in Qom, Iran. Almost 11 days before hospitalization, she had experienced fever and trembling, pain in the left-side of the chest, and coughing with sputum, followed by hemoptysis. She had experienced no weight loss or night sweats. However, she had a history of rheumatoid arthritis with prednisolone treatment.

The patient was admitted to the hospital with a diagnosis of pneumonia while she was experiencing massive hemoptysis. Chest and paranasal computed tomography (CT) scans were applied for the patient, in addition to supportive care. Commonly, evaluation of hemoptysis is carried out by CT scan (3), especially in the case of unexplained hemoptysis (11).The CT scan results showed a heterogeneous and dense lesion in the posterior basolateral side of the left lung. The most probable finding was an expanded lesion. Accordingly, bronchoscopy and CT-guided biopsy were recommended. The differential diagnoses included a fungal hydatid cyst or diaphragmatic hernia. Next, a CT-guided biopsy was carried out. The pathology results showed a lesion with fibrinoid necrosis, suspected of granulomatous inflammation, with a negative acid-fast bacilli (AFB) smear; therefore, TB was suspected in the patient. On the other hand, other diagnostic tests, including direct sputum AFB smear and culture, did not confirm TB or a fungal infection. Nonetheless, anti-TB treatments were initiated, and the patient was transferred to a specialized hospital in Tehran for complementary diagnostic measures, treatment, and follow-up.

As soon as the patient was admitted to the infectious ward of our hospital, bronchoscopy and bronchoalveolar lavage (BAL) were performed. There was no end luminal lesion, and the lavage sample did not indicate any malignancies or infections. Also, six sputum samples were negative for the AFB smear and culture. The serological investigation for a hydatid cyst showed the low titer of anti-hydatid antibodies, which was not clinically significant.

During the patient’s hospitalization in the infectious ward, she experienced massive hemoptysis repeatedly, which was controlled by supportive care. About 16 days after hospitalization in the infectious ward, she was transferred to the surgery ward because of recurrent bleeding and then to the intensive care unit (ICU). It should be noted that surgery has been recommended for treating massive hemoptysis since 1974 (12).

After surgery, the lesion was sent to the pathology department for complementary diagnostics. The patient was under intensive care after surgery, and conservative care was delivered until reaching a stable status. She was in a good general condition upon discharge and was prescribed anti-inflammatory and prophylactic antibiotics.

**Figure 1.**
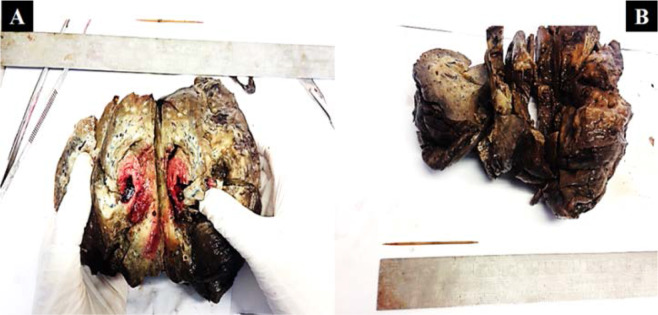
Lung lesion and its size compared with toothpick removed from it

**Figure 2.**
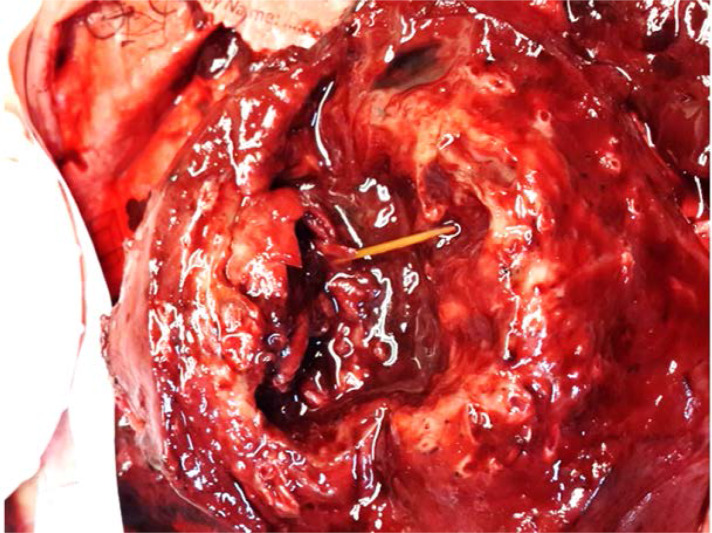
The position of toothpick before pulling-out from lung lesion during gross inspection on frozen section

A lobectomy specimen was obtained for intraoperative consultation. The lesion had a length and width of around 18 cm and 16 cm, respectively (Figure 1). A woody structure was found in the lung, which was found to be a toothpick (Figures 1 & 2). The toothpick structure was normal in terms of form and size (length, 6 cm). Figure 1 presents a comparative view of the lung lesion and the toothpick. Figure 2 also shows the position of the toothpick before being removed.

**Figure 3.**
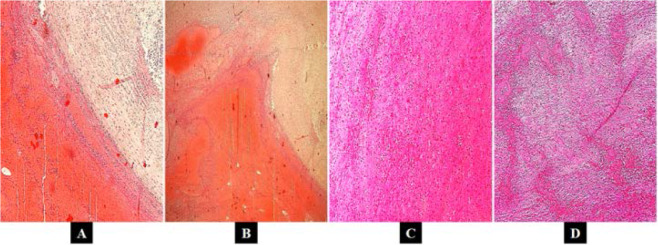
Hematoma and resulted fibrinoid necrosis and fibrosis in the lesion due to the chronic inflammatory responses by the time, after entrance of an unconventional object, the toothpick; A and B, show the hematoma lesion surrounded by the dens fibrous tissue; C and D, show that the center of hematoma is consisted with fibrin.

**Figure 4.**
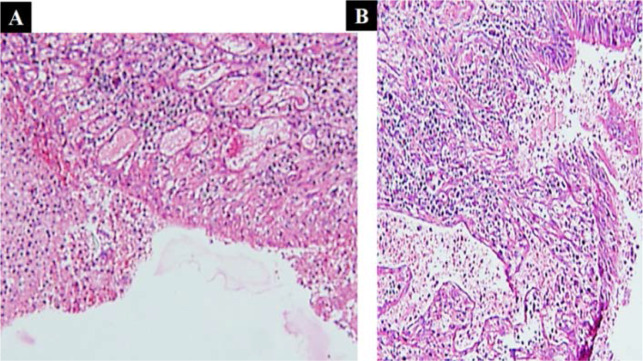
Bronchial wall with ulceration and granulation tissue formation. A and B, infiltration of immune cells is obvious.

**Figure 5.**
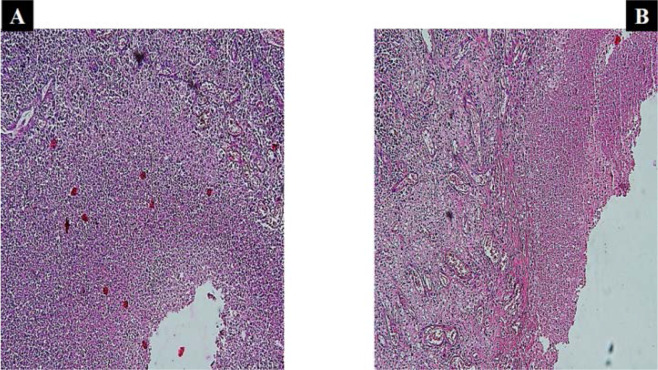
Necrotic debries due to the inflammatory cell reactions after entrance of extranal object, the toothpick. A: magnification is ×40; B: magnification is ×20.

**Figure 6.**
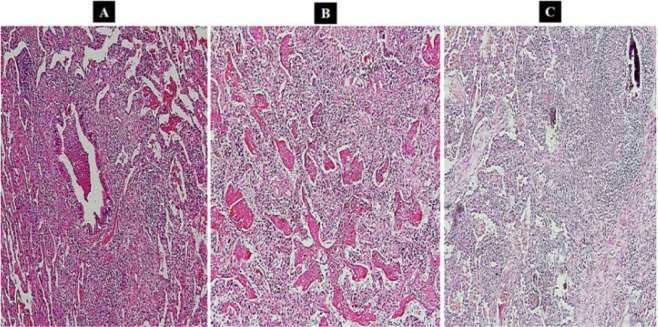
Granulation tissue plugs in lung parenchyma distal to the endobronchial hematoma formation; these plugs are composed predominantly from fibrin. A, B and C: magnification rate is ×40, ×100 and ×200, respectively.

Microscopic investigation revealed ulceration, hematoma, fibrinoid necrosis, and granulation tissue formation due to foreign body aspiration into the lung, resulting in secondary reactions (Figure 3). The aspiration of the toothpick caused ulcers in the bronchial walls and affected them through time, as mentioned earlier. Figures 4, 5, and 6 present the morphological changes.

## DISCUSSION

Foreign body aspiration can lead to severe complications. Aspirated foreign bodies may be organic, such as nuts, seeds, food, and bones. Also, inorganic foreign bodies, which are commonly aspirated, include beads, coins, pins, small parts of toys, and pen caps in children and dental prostheses, pills, and tabs of beverage cans in adults. Both types of aspirated foreign bodies, whether organic or inorganic, can cause inflammatory responses and airway changes (10). Fibrinoid necrosis and granulation formation occur when the foreign body remains in the airway space and can be diagnosed after lung biopsy (13).

In this regard, Misra and Dietl reported massive hemoptysis after the aspiration of a toothpick in a 42-year-old man. The patient was treated via lobectomy after removing a 7-cm toothpick (14). Except for the method of diagnosis and the patient’s gender, most other aspects were similar between our case and the case reported by Misra and Dietl. Nevertheless, we presented more detailed histopathological data about the injured lung tissue. Also, Misra and Dietl identified the toothpick after a CT scan, whereas we found a woody structure after surgical resection and lobectomy. To the best of our knowledge, this report of toothpick aspiration into the lung lobe, resulting in massive hemoptysis, is one of the rare case reports on this type of inflammatory involvement. We believe that the CT scan can be helpful in the diagnosis of foreign body aspiration, but is not adequate, and other investigations are necessary, as shown in our rare case.
